# Hematological responses to iron-folate supplementation and its determinants in pregnant women attending antenatal cares in Mekelle City, Ethiopia

**DOI:** 10.1371/journal.pone.0204791

**Published:** 2018-10-01

**Authors:** Ezra Belay, Asrat Endrias, Birhane Alem, Kedir Endris

**Affiliations:** 1 Department of Medical Biochemistry, College of Health Science, Mekelle University, Mekelle, Ethiopia; 2 Departments of Anatomy, College of Health Science, Mekelle University, Mekelle, Ethiopia; 3 Deparment of Nursing, College of Health Science, Mekelle University, Mekelle, Ethiopia; University of New Mexico, UNITED STATES

## Abstract

**Objective:**

To characterize anemia and evaluate hematological responses to universal iron-folic acid (IFA) supplementation in Ethiopian pregnant women.

**Method:**

A hospital- based prospective follow up study was done between December 2016 and June 2017. Hematological profiles were measured in pregnant women before and after a minimum of one-month IFA supplementation. Mean values and abnormal proportions of hematological profiles were compared before and after supplementation using paired t-test and McNemar test, respectively. Univariate and multivariate analysis were used to analyze the association between independent variables and poor treatment responses.

**Result:**

Lack of adequate hemoglobin response was found in 48.5%(95/196) of the participants. Prevalence of anemia and low hematocrit value were decreased significantly after IFA supplementation (p = 0.002, and p = 0.001, respectively). Normocytic hypochromic anemia was the commonest form of anemia found in this study followed by normocytic normochromic anemia. There was no statistically significant association between poor hemoglobin responses and all studied factors such as educational level, household size, parity, recent illness, stage of pregnancy, coffee consumption, and duration of iron treatment.

**Conclusion:**

Our study revealed poor hemoglobin responses in nearly half of the study participants and a high proportion of anemias morphologically atypical of iron deficiency anemia. There is a need to consider anemia attributable to etiologies other than an iron deficiency in anemia intervention programs.

## Introduction

Anemia during pregnancy is a significant health problem in Ethiopia with a prevalence rate as high as 30% [1]. It poses a major threat to maternal and child survival, contributing to poor pregnancy outcomes, poor cognitive/physical development, and decreased work productivity [2, 3]. A large body of evidence suggested that anemia in pregnancy has an adverse effect on fetal growth which is an important predictor of children’s immediate and future health [4–7]. In combination with obstetric hemorrhage, anemia during pregnancy is estimated to be responsible for 17–46% of cases of maternal death in Africa [8]. Moreover, some studies revealed that anemia could play a secondary role in nearly all maternal cases in resource- poor setting [9].

According to the World Health Organization (WHO) anemia in pregnancy is defined as hemoglobin (Hb) levels < 11.0 g/dl in the first and third trimester of pregnancy and < 10.5 g/dl in the second trimester of pregnancy. However, a cut off of 11.0 g/dl has been used for anemia independent of gestation in most circumstances [2]. There are two classification systems for anemia; based on red cell size (morphological) and underlying pathologic mechanisms. Using red cell size as a criterion, anemia can be classified as microcytic, normocytic, and macrocytic anemia. In microcytic anemias, with a mean corpuscular volume (MCV) less than normal (<80 femtoliters (fL)), iron deficiency or malabsorption, thalassemia and other genetic defects in hemoglobin synthesis are the major etiologies. Macrocytic anemia occurs when mean MCV is above normal level(>100 fL), and are mainly caused by Vitamin B12 and folate deficiency, and Liver Diseases. Normocytic anemia (MCV 80–100 fL) is majorly due to anemia of chronic infection and inflammation, multiple causes and hemolysis or blood loss. Based on underlying mechanism anemia is classified as hypo proliferative (decrease in RBC production) or maturation abnormalities (increase in RBC loss or destruction) [10–12].

In economically poor women, multiple anemia etiologies can coexist due to the poor quality of life and poor diet [13–15]. High prevalence of micronutrient deficiencies that have roles in hemoglobin synthesis such as vitamin A, vitamin B12, folic Acid, riboflavin (vitamin B2) has been reported in isolated studies in Ethiopian pregnant women [16–17]. When multiple micronutrient deficiencies coexist in the same individual, it is possible that a deficiency of one micronutrient influences the etiology, treatment of another micronutrient deficiency [18].

Despite the presence of such complex etiological factors of anemia in resource-poor setting, anemia control programs have been focusing on iron deficiency. Supplementation of iron tablets with or without folate is the most widely used approach to control the global problem of anemia [2]. In Ethiopia, daily iron supplementation for at least 6 months during pregnancy and 3 months postpartum is an integral part of Antenatal Care (ANC) services[19]. Iron- folic acid (IFA) supplementation has been shown to reduce the risk of maternal anemia, maternal mortality, the risks of premature birth and low birth weight [20]. However, recent studies reported the failure of IFA supplementation to restore hemoglobin concentrations to normal [21] and certain populations do not benefit from universal iron supplementation at all [22]. In low resource-setting, anemia is more likely to have complex, multiple causes. In these situations, supplementation normalizes iron stores, whereas low hemoglobin concentrations persist indicating that other micronutrient deficiencies are limiting hemoglobin response to iron supplements [23,24].

Due to limited laboratory facilities, hemoglobin concentration is frequently used as a tool for testing anemia. However, hemoglobin measurement alone cannot determine the cause of the anemia. As a result, the specific underlying causes of anemia and their relative contributions cannot be determined from the available data in Ethiopia, making the diagnosis of the true etiological factor difficult. The aim of this study was to characterize anemia, and the changes in hematological profiles in pregnant women receiving IFA supplementation using RBC morphology and treatment response as a tool.

## Methods and materials

Ahospital-based prospective follow up study was done in two government hospitals found in Mekelle city, Northern Ethiopia from November 2016 to June 2017.

All pregnant women with low initial hemoglobin level (<12 g/dl) who were eligible to start iron-folate supplementation, agreed to give informed consent and have had regular antenatal care follow up at the two hospitals during the study period were included. All non-pregnant women, pregnant women with initial hemoglobin value ≥12 g/dL, and those who failed to give informed consent agreement were excluded from the study. Also participants with comorbid conditions known to affect hemoglobin levels such as intestinal parasite and malaria infection, and chronic gastritis were not eligible for the study.

### IFA supplementation (intervention)

Eligible pregnant women were given 60 mg iron plus 400 μg folic acid oral tablets daily. All pregnant women were using the same type of IFA tablet. A minimum of one-month duration was required before the second sample collection. After one month of supplementation, all hematological profiles are expected to be corrected if the underlying cause for low hemoglobin was iron or folate deficiency. Based on WHO guideline, it is generally recommended for pregnant women to take IFA supplement for six months to fully correct the depleted iron storage.

### Data collection

Data were collected from the study participants in two phases; before and after IFA supplementation. During their first antenatal care visit, the first phase data were collected from eligible pregnant women for the following variables: socio-demographic (age, marital status, education, household size, occupation), health and obstetric history (gestational age, gravidity, live births, history of abortion), recent medical illnesses, food choice, coffee and tea consumptions, complete blood count analysis: red blood cell count (RBC), hemoglobin concentration (Hb), hematocrit(Hct), white blood cells (WBC) count and platelets counts), RBC indices; (mean cell volume (MCV), Mean Corpuscular Hemoglobin (MCH), Mean Corpuscular Hemoglobin Concentration (MCHC). Then, in the second antenatal care visit (after a minimum of one month IFA supplementation), second phase data were collected from the same study participants on the duration of IFA supplementation, treatment adherence, and hematological profiles (Hb, RBC, Hct, MCV, MCH, MCHC, WBC and platelets). There was at least one month gap between the first and the second visits. This was in line with the routine practices of the clinics.

### Hematological analysis

Following a standard and safety collection procedure, 5ml of venous blood sample was collected by senior laboratory technologist with heparinized test tube at Ayder Referal Hospital Central Laboratory and Mekelle general hospital laboratory for hematological analysis. All hematological parameters were measured by an automated analyzer at Ayder referral hospital hematology lab (system XT-4000i). The automated analyzer measures the numbers as well as types of cells available in the blood when they pass through narrow tubes containing sensors, capable to calculate and differentiate the numbers of cells passing through it. Light detectors are used for the measurement of electronic impedance during passage through the hematology analyzer.

### Operational definitions

Anemia was defined as hemoglobin value less than <11g/dl according to world health organization (WHO) criteria [21]. Hemoglobin responses to the universal IFA supplementation were categorized as good or poor/inadequate response based on the following criteria. The increment of hemoglobin value by at least 1g/dl after a minimum of one month supplementation was considered as a good response and indicative of true iron deficiency. On the other hand, a change in hemoglobin value by less than 1g/dl after a minimum of one month IFA supplementation was classified as poor or inadequate response and suggests functional iron deficiencies [11, 25]. The normal reference ranges used for the other hematological parameters were MCH: 27–32 pictogram (pg)/cell; MCHC: 31.5–36 g/dl; MCV: 80–100 fL; Hct: 31–43; RBC count: 3–4.5 x10^12^ cells/L, WBC count: 4-15X 10^9^ cells/L and platelets: 150-450x10^9^/L [26, 27]. Values above or below these reference ranges were classified as high or low, respectively. Anemia was classified morphologically as macrocytic, microcytic, hypochromic and mixed types based on MCV and MCH and/or MCHC values. Gestational ages were also categorized as the first trimester (1–14 weeks), second trimester (5–28 weeks) and third trimester (29 and above) using WHO classification criteria.

Adherence to IFA supplementation was assessed using self- reports by asking pregnant women on how often they took the IFA tables per week in the preceding month. Accordingly, those who had taken IFA tablets at least 4 times per week were considered as adherent. Those who missed one or both IFA tablets three or more times 4 per week were categorized as non-adherent and were excluded from the study. Fourteen participants were excluded because of non-adherence to IFA supplementation based on this criteria.

### Statistical analysis

Data were analyzed using SPSS software version 20.0. Independent variables were categorized or grouped for univariate analysis and displayed in the frequency table. The change in hemoglobin values was calculated by subtracting the baseline measures from the values measured after one or two months of IFA supplementation. All categorical variables were tested for their association with poor hemoglobin responses after IFA supplementation. Categorical variables with a p-value of 0.25 or less in univariate analysis were entered into multivariate analysis after checking co-linearity test. Mean values of hematological profiles before and after supplementation were compared for significant difference using a paired t-test. The significance of differences in proportions (percentages) of abnormal hematological profiles before and after IFA supplementation was checked using the McNemar test. A p-value of <0.05 was considered as indicative of a statistically significant difference.

### Ethical approval

Ethical clearance for the study (ERC 0874/2016) was obtained from Health research ethics review committee (HRERC) of College of Health Sciences, Mekelle University. Subsequent permission was obtained from the authorities of Ayder Referral Hospital and Mekelle general hospital, including medical directors and the heads of maternal clinics. After explaining the objectives and procedures of the study, verbal and written informed consent was obtained from each participant.

## Results

### Characteristics of study participants

Data from 196 pregnant women were included in the final analysis. The mean age of the pregnant women was 25.74, ranged from 16–40 years. A large proportion of the study participants (42.9%) were in ages between 21 and 25 years. While nearly half of the women (49%) had completed their secondary education and 3% of the participants were illiterate. Majority of them (69.4%) had a household size less than three. Nearly one third of the participants (32.1%) were in their first pregnancy. More than half of the women (54.1%) had 1 or 2 children. Thirty four participants had a history of abortion in their previous pregnancy and 31 had a.history of medical illness in the previous six months. These illness were urinary tract infection (6/196), loss of appetite(8/196), hepatitis B virus infection (3/196), typhoid (4/196) and others (10/196).Majority of the pregnant women (53.6) were in their third trimester while visiting the antenatal care for the first time. Only 5% of the women were in their first trimester during their first antenatal care visit. More than half of the pregnant women included in the study (52.0%) took IFA supplementation for 3 to 4 months. Most of the participants (80.2%) drink coffee every day (Table 1).

**Table 1:** Sociodemographic characteristics of pregnant women attending antenatal cares at Mekelle and Ayder Referral hospital, Mekelle, June 2017.

	Response to IFA supplementation		COR(95%CI)	p-value
Characteristics	Good response (N = 101), n(%)	Inadequate response (N = 95), n(%)		
Age, mean± SD	25.7±4.4	25.7±4.6		
≤20 years	10(9.9)	13(13.7)	Reference	
21–25 years	44(43.6)	40(42.1)	0.7(0.28–1.77)	0.7
26-30years	33(32.7)	31(32.6)	0.7(0.28–1.87)	0.7
>30 years	14(13.9)	11(11.6)	0.6(0.19–1.9)	0.6
Education				
Illiterate	2(2.0)	4(4.2	2.8(0.46–17.2)	0.3
Primary	26(25.7)	27(28.4)	1.5(0.64–3.34)	0.4
Secondary	49(48.5)	47(49.5)	1.4(0.6–2.8)	0.4
Above Secondary	24(23.8)	17(17.9)	Reference	
Household size				
≤3	71(70.3)	65(68.4)		R
>3	30(29.7)	30(31.6)	1.1(0.6–2.01)	0.8
*Occupation			Reference	
Government	60(59.4)	46(48.4)		
Laborer	10(9.9)	16(16.8)	2.1(0.86–5.0)	0.1
Merchant	21(20.8)	25(26.3)	1.6(0.7–3.1)	0.2
Others	10(9.9)	8(8.4)	1.1(0.38–2.85)	0.9
*Parity				
0	38(37.6)	25(26.3)	Reference	
1–2	51(50.5)	55(57.9)	1.6(0.8–3.1)	0.13
> = 3	12(11.9)	15(15.8)	1.9(0.7–4.7)	0.17
*Stage of pregnancy				
First trimester	7(6.9)	3(3.2)	Reference	
Second trimester	44(43.6)	37(38.9)	1.9(0.4–8.1)	0.4
Third trimester	50(49.5)	55(57.9)	2.6(0.6–10.5)	0.2
History of Abortion, Yes	17(16.8)	17(17.9)	1.1(0.5–2.26)	0.8
recent illnesses, Yes	15(14.9)	16(16.8)	1.1(0.5–2.3)	0.9
Duration IFA supplmentation				
1–2 months	46(45.5)	48(50.5)	1.2(0.7–2.14)	0.5
3–4 months	55(54.5)	47(49.5)	Reference	
coffee drinking, Yes	81(80.2)	81(85.3)	1.5(0.6–3.0)	0.4
Time of coffee				
Before meals	20(19.8)	20(21.1)	Reference	
After meals	54(53.5)	48(50.5)	0.9(0.42–1.81)	0.8
No time preference	7(6.9)	13(13.7)	1.9(0.6–5.63)	0.3

SD: standard deviation; R: reference group; COR: crude odd ratio; CI: confidence interval

* variables that were entered to multivariate analysis.

### Hematological responses to IFA supplementation

Hemoglobin response was measured in pregnant women with Hb value less than 12 g/dl. Accordingly, adequate Hb response, increment by 1g/dl and above, was found only in 51.5% of the pregnant women taking IFA. There was no adequate hemoglobin response in the rest 48.5% of study participants. Before IFA supplementation, a quarter of pregnant women (25.5%) were diagnosed with anemia (Hb<11.0 g/dl). After supplementation, this was dropped to 13.8%(p = 0.002). Fifteen women who were diagnosed with anemia in their first visit remained anemic after IFA supplementation. The other 11 women, who were non-anemic before supplementation, became anemic after IFA supplementation. A large proportion of the pregnant women had low MCHC (64.3%), MCH (25.5%), and MCV (18.4%) on their first visit before IFA treatment and remains nearly unchanged after IFA supplementation. Only 3.6% of them have had low WBC and platelet counts before treatment and was changed into 1.5% and 5.6%, respectively after treatment. Except for low hemoglobin and hematocrit values, the changes in the proportions of all other abnormal hematological profiles were not statistically significant after IFA treatment (Table 2).

**Table 2:** Proportions of pregnant women with an abnormal hematological profile before and after IFA supplementation at Mekelle and Ayder Referral hospital, Mekelle, June 2017.

Abnormal hematological profiles	Before treatment: n(%)	After treatment:n(%)	p-value
Anemia (Hb<11g/dL)	50(25.5)	27(13.8)	0.002
Low Hct(<31)	20(10.2)	5(2.6)	0.001
High MCV(>100fl)	13(6.6)	16(8.2)	0.61
Low MCV(<80fl)	36(18.4)	46(23.5)	0.09
Low MCH(<27pg)	49(25.0)	44(22.4)	0.83
Low MCHC(<32g/dL)	140(71.4)	73.0(76.5)	0.24
Low WBC count (<4x10^9^cells/L)	7(3.6)	3(1.5)	0.38
Low PLT (<150x10^9^platelets/L)	5(2.6)	11(5.6)	0.55

Hb: hemoglobin, Hct: hematocrit, RBC: Red Blood Cells count, MCV: Mean Cell Volume, MCH: Mean Corpuscular Hemoglobin, MCHC: Mean Corpuscular Hemoglobin Concentration, WBC: White Blood Cell Count, PLT: Platelet; p-values were obtained from McNemar test.

The mean hemoglobin value was changed from 10.4 to 11.5 in anemic (10.6% increment) and from 11.5 to 12.5 in non-anemic pregnant women (8.7% increment) after IFA supplementation. The mean hematocrit value was also increased by 13% in anemic women and by 9.7% in non-anemic pregnant women. The changes in mean hemoglobin and hematocrit values after IFA supplementation was statistically significant (P = 0.001) in both groups. The mean MCV value was declined from 87.1 to 81.7 (decreased by 6.2%) in anemic women and from 85.1 to 83.7 (dropped by 1.7%) in non-anemic groups. Similarly, the mean MCH, MCHC and platelet values were decreased by 1.1%, 2.2%, and 2.5% after supplementation in anemic groups. But the mean RBC and WBC counts were increased by 2.3 and 1.4%in these groups (p>0.05) (Table 3). Different trends were observed in non-anemic groups. While the mean MCHC value and WBC count were decreased by 0.97% and 4.1%, respectively, the mean MCH value and platelet count were increased by 1.3% and 1.7%, respectively after supplementation. Except for hemoglobin, hematocrit in both groups, and MCH in non-anemic groups, the changes in the mean value of all other hematological profiles were not statistically significant (p>0.05)(Table 3).

**Table 3:** Changes in the mean values of hematological profiles after IFA supplementation in anemic and non-anemic pregnant women attending antenatal cares at Mekelle and Ayder Referral Hospital, Mekelle, June 2017.

Hematological responses to IFA Supplementation in anemic pregnant women					
Parameters	mean ± SD Before supplementation	mean ±SD After supplementation	Mean difference (95% CI)	% change	p-value
Hemoglobin	10.4±0.5	11.5±1.3	1.1(0.8–1.45)	10.6	0.0001
Hematocrit	32.3+2.8	36.6±3.9	4.3(3.1–5.6)	13.3	0.0001
RBC	4.4±1.2	4.5±1.2	0.1(-0.02–0.2)	2.3	0.09
MCV	87.1±7.5	81.7±22.5	-5.4(-11.5–0.6)	-6.2	0.08
MCH	27.8±2.8	27.5±3.0	-0.3(-0.72–0.1)	-1.1	0.19
MCHC	31.0±2.5	30.3±2.5	-0.7(-1.4–0.2)	-2.2	0.16
WBC	7.1±1.9	7.2±2.0	0.1(-0.5–0.6)	1.4	0.84
PLT	306.5±138.6	298.8±67.1	-7.7(-45.7–30.3)	-2.5	0.68
Hematological responses to IFA supplementation in non-anemic pregnant women(Hb<12g/dL)					
Hemoglobin	11.5±0.3	12.5±1.2	1.0(0.8–1.2)	8.7	0.001
Hematocrit	35.2±2.8	38.6±3.3	3.3(2.6–3.9)	9.7	0.001
RBC	4.8±1.5	4.9±1.5	0.1(-0.04–0.1)	2.0	0.27
MCV	85.1±11.7	83.7±19.6	-1.4(-4.6–1.9)	-1.7	0.41
MCH	30.2±8.4	30.6±8.4	0.4(.05–0.7)	1.3	0.03
MCHC	31.0±2.9	30.7±2.8	-0.3(-0.6–0.03)	-0.97	0.06
WBC	7.3±2.2	7.0±2.0	-0.2(-0.6–0.1)	-4.1	0.12
PLT	303.9±64.9	309.1+69.2	5.2(-4.3–14.8)	1.7	0.3

RBC: Red Blood Cells count, MCV: Mean Cell Volume, MCH: Mean Corpuscular Hemoglobin, MCHC: Mean Corpuscular Hemoglobin Concentration, WBC: White Blood Cell Count, PLT, Platelet.;p-values were obtained from paired t-test

### Anemia morphology and classification

From 50 anemias diagnosed in the first visit, 56% (28/50) were morphologically mixed type (normocytic hypochromic anemia) with normal MCV (80–100 fl) and low MCH (<27pg) and or MCHC (<31.5g/dl). Only 5 of them were microcytic hypochromic, typical of iron deficiency anemia, with low MCV (<80fl) and low MCH (27 pg) and/ or MCHC (<31.5g/dl). The remaining 28% (14/50) and 6% (3/50) were normocytic normochromic and macrocytic anemias, respectively (Table 4).

**Table 4:** Anemia classifications based on red blood cell indices in pregnant women attending antenatal cares at Mekelle and Ayder Referral hospital, Mekelle, June, 2017.

Anemia morphology	Before IFA supplementation (n = 50)	After IFA supplementation (n = 27)
Normocytic normochromic (normal MCV,MCH and MCHC)	14	12
Microcytic hypochromic (low MCV,MCH,MCHC)	5	7
Macrocytic (high MCV)	3	-
Mixed morphology (normocytic hypochromic)	28	8

MCV: Mean Cell Volume, MCH: Mean Corpuscular Hemoglobin, MCHC: Mean Corpuscular Hemoglobin Concentration

### Determinants of poor hemoglobin response after IFA supplementation

In bivariate analysis variables including age group, educational level, occupation, number of households, parity, stage of pregnancy, abortion history, other illnesses, duration of IFA supplementation, coffee consumption and time coffee consumption, were tested for their association with poor hemoglobin response after IFA supplementation. Unfortunately, none of these variables showed statistically significant association with poor hemoglobin responses (Table 1). Though not statistically significant, illiterates, laborers, pregnant women with 3 or more gravida, those who were on their third trimester, and those who were drinking coffee without time preference were more likely to have poor hemoglobin responses(Table 1). Variables with P<0.25 in bivariate analysis, including occupation, parity and stage of pregnancy, were entered into multivariate analysis but none of them revealed statistically significant association.

## Discussion

Though several studies have determined the prevalence of anemia in Ethiopian pregnant women, data on specific anemia etiology is still limited. On the other hand, recent studies revealed that magnitude of anemia attributable to iron deficiency varied from region to region and several other factors could play a major role in low hemoglobin and anemia [28]. In this study, we tried to characterize anemia, and identify its causes using hematological responses to universal IFA supplementation and RBC morphology as a tool.

Our study revealed that hemoglobin response to IFA supplementation was inadequate or poor in 48.5% of the study participants. This was slightly higher than the reports of Tahaineh et al from Jordan (43.1% non-response rate) [29]. Since we included patients that were adherent and consumed IFA supplementation for at least one month, non-compliance and short duration are not the likely causes for the failure of hemoglobin response. Instead, lack of response to IFA supplementation could be attributable to other etiologies or functional iron deficiencies. While absolute iron-deficient anemia promptly responded to IFA supplementation, anemia of chronic infections or inflammation and multiple causes such as combined micronutrient deficiencies lead to functional iron deficiencies which impair iron utilization [30, 31]. Iron for erythropoiesis in bone marrow is mainly obtained from re-use of iron from senescent RBCs and other cells by macrophages, dietary/supplement iron absorption in the duodenum, and release of stored iron from hepatocytes [30]. Chronic infections/inflammations and combined micronutrient deficiencies obstruct cellular iron export to extracellular fluid for utilization by erythroid precursors in bone marrow, restricting iron recycling and then impairing erythropoiesis [32, 33].

Similarly, the morphology of the anemias found in this study was suggestive of functional iron deficiency or multiple causes [34]. Accordingly, normocytic hypochromic anemia was the commonest pattern of anemia in the study participants followed by normocytic normochromic anemia. Hypochromic erythrocytes with low MCH (25% and/or MCHC (71.4%), which are indicators of ineffective hemoglobinization of erythrocytes as a result of functional iron deficiency[35], were common. During anemia of chronic infections, inflammations, and concurrent micronutrient deficiencies, iron stores are adequate, but there is impaired iron delivery to the developing red cell. As a result, the hemoglobin content of newly synthesized RBC will be decreased (hypochromic) [10]. However, normocytic hypochromic anemia was significantly lowered after supplementation, from 28/50 to 8/27. This may due to either lack of specificity in MCV measurement or morphologic changes that occur over the time. As several possible factors can cause this morphologic abnormality, the morphologic of RBC can be changed as the causes are altered overtime [10]. Therefore, this may not be necessarily due to the effect of supplementation alone. Our results also indicated that IFA supplementation improved neither the mean values of MCH and MCHC nor the proportion of pregnant women with low MCH and MCHC. On the other hand, the proportion of normocytic normochromic anemia remains almost unchanged after supplementation in our study. As this type of anemia is characteristics of functional iron deficiencies, supplementation may not improve it.

The proportion of microcytic hypochromic anemia, which is an indicator of iron deficiency anemia, thalassemia and sideroblastic anemia was not changed after supplementation. In fact, the proportion was slightly increased from 5/50 to7/27 anemic pregnant women. The main reasons for this may be either malabsorption and the presence of concurrent micronutrient deficiencies such as vitamin A or due to other genetic defects in heme synthesis that can cause the same RBC morphology. In agreement with this, previous studies found a better hemoglobin response in pregnant women receiving IFA supplementation when other micronutrients such as vitamin A were included [36]. Generally, it is believed that iron deficiency is the most common cause of anemia (~50%) during pregnancy and has been the main focus of anemia control programs in pregnant women in Ethiopia. In contrast, our RBC morphology and IFA treatment response data do not support this general notion. Instead, only small proportions of anemia (~10%) attribute to iron deficiency. This was also in line with findings of Gebreegziabher and Stoecker in southern Ethiopian women of reproductive ages. Using multiple iron biomarkers, the authors found only 5% of iron deficiency anemia from 21.5 total anemic cases identified, supporting that iron deficiency may not be a major contributor of anemia in Ethiopia pregnant women as it is predicted before[37].This suggests the need to consider anemia attributable to factors other than an iron deficiency in designing effective public health intervention program for anemia control. On the other hand, large doses of iron supplements in those who are not in real iron deficiency may lead to iron overload in pregnant women. Iron overloading following supplementation can induce production of free radicals and oxidative damage via Fenton reactions. This in turn associates with the initiation of various pathogenic processes such as cardiovascular disease, neuropathologies, and cancer [38]. Moreover, studies have shown that iron overload is associated with poor clinical outcomes in infectious diseases [28].

We also evaluated the different sociodemographic, maternal and clinical factors that can contribute to poor hemoglobin responses to IFA supplementation. Nevertheless, there was no statistically significant association between poor hemoglobin responses after treatment and studied factors such as educational level, household size, and parity, the presence of other illness, stage of pregnancy and, coffee consumption. Though coffee is recognized as an inhibitor of non-heme iron absorption [39], we do not find significant association between coffee consumption and failure to respond treatment. But those who consumed iron without time preference were more likely to have poor responses. Pregnant women started their iron treatment lately in their second and third trimesters had more risks of failure to respond to the treatment. Similarly, pregnant women with 3 or more gravidity were also more likely to have poor responses. This was in line with previous studies’ report on the impact of parity and gravidity on severity anemia [40].

This study has some limitations. Firstly, we did not measure the serum value of other micronutrients such as zinc, vitamin A, B vitamins and inflammatory markers that could affect treatment responses. Secondly, it could have been better if we monitored serum iron parameters such ferritin level at baseline and after treatment along with hematological responses to get further information on iron bioavailability and rule out the different causes of anemia. Finally, longer follow up studies on IFA supplementation response and effect starting from the first trimesters could be important to monitor the significance of the supplementation program in the local population.

## Conclusion and recommendation

Our study revealed that poor hemoglobin responses in nearly half of the pregnant women participated in the study and a high proportion of anemias morphologically atypical of an iron deficiency anemia. There is a need to consider anemia attributable to etiologies other than iron deficiency in anemia intervention programs. The current universal iron supplementation program should be reassessed for possible changes into targeted supplementations.
