# Correction: Hematological responses to iron-folate supplementation and its determinants in pregnant women attending antenatal cares in Mekelle City, Ethiopia

**DOI:** 10.1371/journal.pone.0212713

**Published:** 2019-02-22

**Authors:** Ezra Belay, Asrat Endrias, Birhane Alem Berihu, Kedir Endris

The third author's name is spelled incorrectly. The correct name is: Birhane Alem Berihu. The correct citation is: Belay E, Endrias A, Berihu BA, Endris K (2018) Hematological responses to iron-folate supplementation and its determinants in pregnant women attending antenatal cares in Mekelle City, Ethiopia. PLoS ONE 13(10): e0204791. https://doi.org/10.1371/journal.pone.0204791
